# Which technique for radiation is most beneficial for patients with locally advanced cervical cancer? Intensity modulated proton therapy versus intensity modulated photon treatment, helical tomotherapy and volumetric arc therapy for primary radiation – an intraindividual comparison

**DOI:** 10.1186/s13014-015-0402-z

**Published:** 2015-04-17

**Authors:** Simone Marnitz, Waldemar Wlodarczyk, Oliver Neumann, Christhardt Koehler, Mirko Weihrauch, Volker Budach, Luca Cozzi

**Affiliations:** Department of Radiation Oncology, Charité University Hospital, Berlin, Germany; Department of Gynecology, Charité University Medicine, Berlin, Germany; Radiosurgery and Radiotherapy Department, Istituto Clinico Humanitas Cancer Center and Reaserch Hospital, 20100 Rozzano, Italy

**Keywords:** Cervical cancer, Intensity-modulated radiotherapy, VMAT, Proton therapy

## Abstract

**Background:**

To compare highly sophisticated intensity-modulated radiotherapy (IMRT) delivered by either helical tomotherapy (HT), RapidArc (RA), IMRT with protons (IMPT) in patients with locally advanced cervical cancer.

**Methods and materials:**

Twenty cervical cancer patients were irradiated using either conventional IMRT, VMAT or HT; ten received pelvic (PEL) and ten extended field irradiation (EFRT). The dose to the planning-target volume A (PTV_A: cervix, uterus, pelvic ± para-aortic lymph nodes) was 1.8/50.4 Gy. The SIB dose for the parametrium (PTV_B), was 2.12/59.36 Gy. MRI-guided brachytherapy was administered with 5 fractions up to 25 Gy. For EBRT, the lower target constraints were 95% of the prescribed dose in 95% of the target volume. The irradiated small bowel (SB) volumes were kept as low as possible. For every patient, target parameters as well as doses to the organs at risk (SB, bladder, rectum) were evaluated intra-individually for IMRT, HT, VMAT and IMPT.

**Results:**

All techniques provided excellent target volume coverage, homogeneity, conformity. With IMPT, there was a significant reduction of the mean dose (D_mean_) of the SB from 30.2 ± 4.0 Gy (IMRT); 27.6 ± 5.6 Gy (HT); 34.1 ± 7.0 (RA) to 18.6 ± 5.9 Gy (IMPT) for pelvic radiation and 26.3 ± 3.2 Gy (IMRT); 24.0 ± 4.1 (HT); 25.3 ± 3.7 (RA) to 13.8 ± 2.8 Gy (IMPT) for patients with EFRT, which corresponds to a reduction of 38-52% for the D_mean_ (SB). Futhermore, the low dose bath (V_10Gy_) to the small bowel was reduced by 50% with IMPT in comparison to all photon techniques. Furthermore, D_mean_ to the bladder and rectum was decresed by 7-9 Gy with IMPT in patents with pelvic radiation and EFRT.

**Conclusion:**

All modern techniques (were proved to be dosimetrically adequate regarding coverage, conformity and homogeneity of the target. Protons offered the best sparing of small bowel and rectum and therefore could contribute to a significant reduction of acute and late toxicity in cervical cancer treatment.

## Background

Concomitant cisplatin-based chemo-radiation in the treatment of locally advanced (uterine) cervical cancer is the standard of care but leads to considerable acute and late toxicity affecting the gastrointestinal (GI) and genitourinary (GU) tracts [1-4]. In patients with para-aortic disease, the use of extended field radiation therapy (EFRT) can be associated with an high incidence of acute and late GI toxicity [5-8].

Intensity-modulated radiotherapy (IMRT) significantly reduces acute and late toxicity to the organs at risk (OAR) [9-11]. A growing body of evidence indicates a strong dose-volume relationship for the development of bowel toxicity. Quantec recommended to allow V_15Gy_ = 120 cc if individual bowel loops are outlined or V_45Gy_ ≤ 195 cc if entire peritoneal potential space of bowel is outlined [12]. Using the constraints of <40% of the bowel should receive at least 35 Gy and <50% of the rectum receiving at least 45 Gy, only 1 out of 54 patients developed grade 3 GI toxicity during an adjuvant chemoradiation trial in cervical cancer [13]. Isohashi et al. [14] demonstrated that the volume of small bowel receiving more than 40 Gy resulted a clear independent predictor of chronic GI complication. Chopra et al. in a prospective study on 71 patients demonstrated the relevance (in multivariate analysis) of the volume of small and large bowels receinving more than 15 Gy. Also the volumes of small bowel receiving 30 and 40 Gy resulted significant in univariate analysis [15].

With its characteristic Bragg peak, proton therapy holds the promise of further reduction of toxicity to organs at risk [16-20]. Different techniques like Intensity Modulated Proton Therapy (IMPT) and passive scattered proton therapy are available. IMPT is progressively being adopted as a standard for proton therapy although the experience with large treatment volumes is still relatively limited.

Intensity modulated protons, with the scanning beam technology, are able to modulate intensities to fulfill dose constraints to the target and the organs at risk. Data from a small study on only five patients with cervical carcinoma suggested a significant decrease of the doses to the kidneys and the femoral heads with protons. Sparing of rectal wall and bladder was superior with protons as well [21]. Another study on ten patients, mainly in the adjuvant situation after surgery, has shown that compared with IMRT alone, the combination of passive scattering protons or intensity modulated protons lead to a statistically significant decrease of dose to the organs at risk, most notably the small and large bowel and kidney, in addition to the bowel and body dose, all while maintaining excellent target coverage [22].

With regard to target parameters all plans were comparable. Dose volume parameters for the bowels were significantly improved with protons compared to intensity modulated photons, with mean dose reductions of 50–80%. The study was limited by the number of patients and the use of outdated 3D-radiation which was compared with the advanced techniques [21]. Only one publication focused on oncologic outcome of brachytherapy emulation with protons and toxicity [23].

Our objective in the present study was to assess whether intensity modulated protons provides any benefit in an intra-individual comparison to all available sophisticated photon techniques with regard to the target and organs at risk for cervical cancer patients undergoing pelvic irradiation or extended field irradiation for locally advanced cervical cancer. The planning goal was to maintain high target coverage while keeping the dose to the bowel as low as possible.

## Materials and methods

### Patient characteristics and treatment planning

Patients’characteristics are shown in Table 1. All patients where treated according to Helsinki declaration; for the present study, being a planning experiment in-silico without actual treatment, data derived from previous treatment were completely anonymised and no further ethical committee approval was needed. All patients received a planning CT scan (CT scanner LightSpeed, GE Healthcare) with intravenous contrast media at a slice thickness of 3.75 mm. In case of para-aortic involvement, the CT scans were done from the diaphragm to the trochanter minor. In all other patients, the CT scan was performed from the 2nd lumbar vertebra to the trochanter minor. The planning CT was performed while the patient was in the supine position using a knee and foot positioning device, and patients were asked to have a full bladder.

**Table 1:** **Patients’ characteristics**

**Parameter**	
Age	Median: 46
	Range: 33-71
FIGO stage	IB2: 2
	IIA: 2
	IIB: 11
	IIIA: 1
	IIIB: 3
	IVA: 1
Grading	G1: 0
	G2: 14
	G3: 6
Histology:	Squamous cell: 16
	Adenocarcinoma: 3
	Adeno-squamous: 1
Treatment volume	Pelvis only (PEL): 10
	Extended field (EFRT): 10
Pelvic lymph nodes	pN0: 4
	pN1: 11
	pNx: 5
Para-aortic lymph nodes	pM0: 7
	pM1: 8*
	pMX: 5**

*All patients underwent extended field radiotherapy; **2/5 pMX patients underwent extended field radiotherapy because of enlarged nodes.

Target volumes and OAR were delineated in all axial CT slices according to the recommendations [24,25]. The planning target volumes were divided into PTV-A and PTV-B (boost) volumes, and the concept of simultaneously-integrated boost (SIB) was applied. The clinical target volume (CTV-A) was defined as the macroscopic tumour, including the cervix and the corpus uteri and external, internal, common iliac, and pre-sacral LNs plus/minus the paraaortic LNs with a 5-mm margin. The volume (PTV-A) was outlined as the CTV-A with a 1-cm margin in all directions (inclusive of set-up and motion related margins, not distinguished between the two as per institutional protocol). In patients with negative para-aortic lymphnodes, the upper field border was at the level of the L4/5 interspace (the pelvis only group, PEL). In patients with para-aortic lymphnode metastases, the para-aortic region was included in the CTV-A up to the level of the renal vessels (the extended field radiotherapy group, EFRT). The caudal PTV slice was at the level of the obturator foramen.

For the SIB target volume defined as CTV-B, the high-risk volume (parametria and surrounding lymphatic tissue) was delineated by titanium clips that were positioned during laparoscopic staging. In patients who did not undergo laparoscopic staging, standardized borders for boost definition were as follows: for the cranial border, the bifurcation of the common iliac artery; for the lateral border, the iliopsoas muscle; for the medial border, the lateral part of the uterus; and for the caudal border, the pubo-coccygeal muscle as part of the levator ani. For the SIB target volume PTV-B, one cm was added to the CTV-B.

The following OARs were delineated: spinal cord, femoral heads, kidneys, bladder, rectum up to the sigmoidal loop, and the bowels as a whole peritoneal cavity except for the lymph nodes, the muscles and the OARs other than the bowels. The delineation of the bowels exceeded the upper border of the PTV-A by 2 slices.

In addition to external beam radiotherapy, these patients underwent Ir-192-HDR-brachytherapy with a total dose of 25 Gy (5 fractions at 5 Gy each) delivered to macroscopic tumour defined on the basis of the MRI image (Gammamed 12i and Brachyvision, Varian Medical Systems, Palo Alto, CA), which was not included in the analysis.

### Dose prescription, planning parameters, and radiation technique

According to the SIB technique, the PTV-A is the difference between the previously assigned PTV-A and PTV-B (SIB target volume). PTV-B is consistent for pelvic and EFRT patients. All dose prescriptions and constraints for the PTV-A, as well as the following analysis, refer to this volume difference unless otherwise noted.

The total dose prescribed for the PTV-A was 50.4 Gy delivered in 28 fractions of 1.8 Gy. The corresponding dose prescribed to the PTV-B was 59.36 Gy delivered in 28 fractions of 2.12 Gy. The lower constraint for both types of PTV was 95% of the prescribed dose in 95% of the target volume, and the upper dose constraint was 107%, which was only reasonable for PTV-B. The bowels volumes receiving 45 Gy (V_45Gy_) and mean dose to the small bowel were to be kept as low as possible without compromising the PTV constraints. Therefore, additional help structures in the form of PTV-free bowels and a 1.5 cm shell were used. Other OAR were also constrained so as not to exceed their critical dose values, but they were considered less important than the bowels.

All plans were optimised by experienced staff members, one per each technique and blindly to each other. This was to ensure homogeneity of quality within a technique, best expertise between techniques and avoid subjective biases as much as possible.

#### Helical tomotherapy planning

Contouring for both IMRT modalities was performed with the Treatment Planning System (TPS) of Eclipse (Varian Medical Systems, Palo Alto, CA). The CT datasets with contoured structures were then transferred to the Tomotherapy TPS (TomoTherapy Inc., Madison, WI), enabling inverse treatment planning for photon irradiation at 6MV with HT. Parameters for beamlet calculation were a field width of 5 cm, pitch of 0.25, and normal resolution mode. The maximum modulation factor we allowed for the optimization was 2.5.

#### Conventional linac-based IMRT planning

Conventional linac-based IMRT plans were calculated for photon irradiation at 20MV using the Eclipse TPS. Also, the TPS enables inverse planning. These plans were generated for an arrangement of seven beams with gantry angles of 45, 90, 115, 180, 245, 280, and 320°. The dynamic (sliding window) technique was used.

#### Rapid arc planning

RapidArc (RA) plans were optimised with the Progressive Resolution Optimisation algorithm in the Eclipse TPS. The optimisation process is based on an iterative inverse calculation that optimizes simultaneously the instantaneous multi-leaf collimator (MLC) positions, the dose rate, and the gantry rotation speed in order to achieve the desired therapeutic dose distribution. Plans were optimised for three full arcs. All dose calculations for IMRT and RA plans were performed with the Anysotropic Analytical Algorithm with a spatial resoluton of 2.5 mm.

#### IMPT planning

IMPT plans have been computed on the Eclipse TPS (v.13) simulating a Varian ProBeam system. A nonlinear universal Proton Optimizer was used for the scope. For the final dose calculation, the Proton Convolution Superposition algorithm was used. A constant RBE of 1.1 was applied. Accuracy of the calculation is discussed for example in [26,27], pointing to a difference between calculated and measured point dose for prostate treatment of 0.0 ± 0.7% and a γ-index of 96.2 ± 2.6%.

The Bragg peak distribution in depth was derived from a range of energies from 70 to 245 MeV. Energy layers were determined to cover proximally and distally the target. Spot spacing was set to 4 mm, circular lateral target margins were set to 5 mm, proximal and distal margins to 5 mm.

For each patient, in the PEL group without inclusion of the pelvic nodes, three beams were used to optimize the plans: two oblique-anterior (OA) fields and one posterior. Gantry angles of the two OA fields were individually chosen to identify the best geometrical setting individually with the aim of minimising the involvement of the bowels. For the patients in the EFRT group, those including the pelvic nodes, two additional anterior-posterior fields were used to cover the more cranial section of the target volumes.

### Dose volume histogram (DVH) analysis

The DVH analysis was applied to both the PTVs and the OARs, respectively. The PTVs were analyzed with regard to dose conformity and homogeneity [28]. One of components for conformity evaluation is the coverage of corresponding target volumes by the prescribed dose, whereas for the SIB target volume PTV-B, the target definition is unambiguous. The main PTV target volume should be the sum of PTV-A and PTV-B, but only throughout the conformity analysis. The Conformity Index (CI) was calculated as the ratio of the volume receiving 95% of the prescribed dose (V_95%_) and the corresponding PTV. The CI definition is useful but fails in cases of insufficient coverage. Therefore, we used the Conformity Number (CN) as an additional conformity criterion, which is a product of the coverage factor (CVF) and the healthy tissue conformity index (HTCI): CN = CVFxHTCI. The CVF is defined as the ratio of the part of the PTV receiving 95% of prescribed dose and the whole PTV. The HTCI is defined as a ratio of two volume parts, the PTV and the total volume, receiving at least 95% of the prescribed dose. Homogeneity (HI) was defined as the ratio of the dose received by 95% (D_95%_) of the volume to the minimum dose received by the “hottest” 5% (D_5%_) of the target volume of interest [29]. The range of ±5% was chosen as a compromise between the ranges of ±10% and ±2% recommended for total body and head and neck irradiation, respectively. Most of the OAR were analyzed by mean dose (D_mean_) or doses to 10% and 90% of the organ volume, respectively (D_10%_, D_90%_).

#### Statistics

Results were analyzed to assess the benefit for each treatment group separately. Data from all plans were compared with the non-parametric Wilcoxon exact signed rank test (SPSS 15.0, Inc., Chicago, IL). Statistical significance was assumed at the level of *p* ≤ 0.05.

## Results

Twenty patients with FIGO stage IB2-IVA with a mean age of 46 years (33–71 years) were treated with primary chemo-radiation. Ten patients underwent pelvic irradiation, only, and ten patients underwent EFRT. All underwent external beam radiotherapy and brachytherapy and received simultaneous chemotherapy with cisplatin 40 mg/m^2^ of the body surface area weekly concomitant with radiation therapy. Patients’characteristics and lymph node stage is shown in Table 1.

The mean volume values for PTV-A were 1297.5 ± 211.8 cc for PEL and 1539.8 ± 334.1 cc for EFRT. The corresponding volume values for the sum of PTV-A and PTVB in the PEL and EFRT groups were 1651.0 ± 290.4 cc and 1835.9 ± 396.8 cc, respectively. Mean volume values for PTV-B did not differ with 353.5 ± 99.4 cc (PEL) and 334.0 ± 130.6 cc (EFRT). The mean volume values for the SB were more than doubled for EFRT with 3283.3 ± 992.8 cc. The median bladder and rectal volume was 212 and 69.2 cc, respectively.

### Comparison of HT , IMPT, IMRT and VMAT

**Figure 1:**
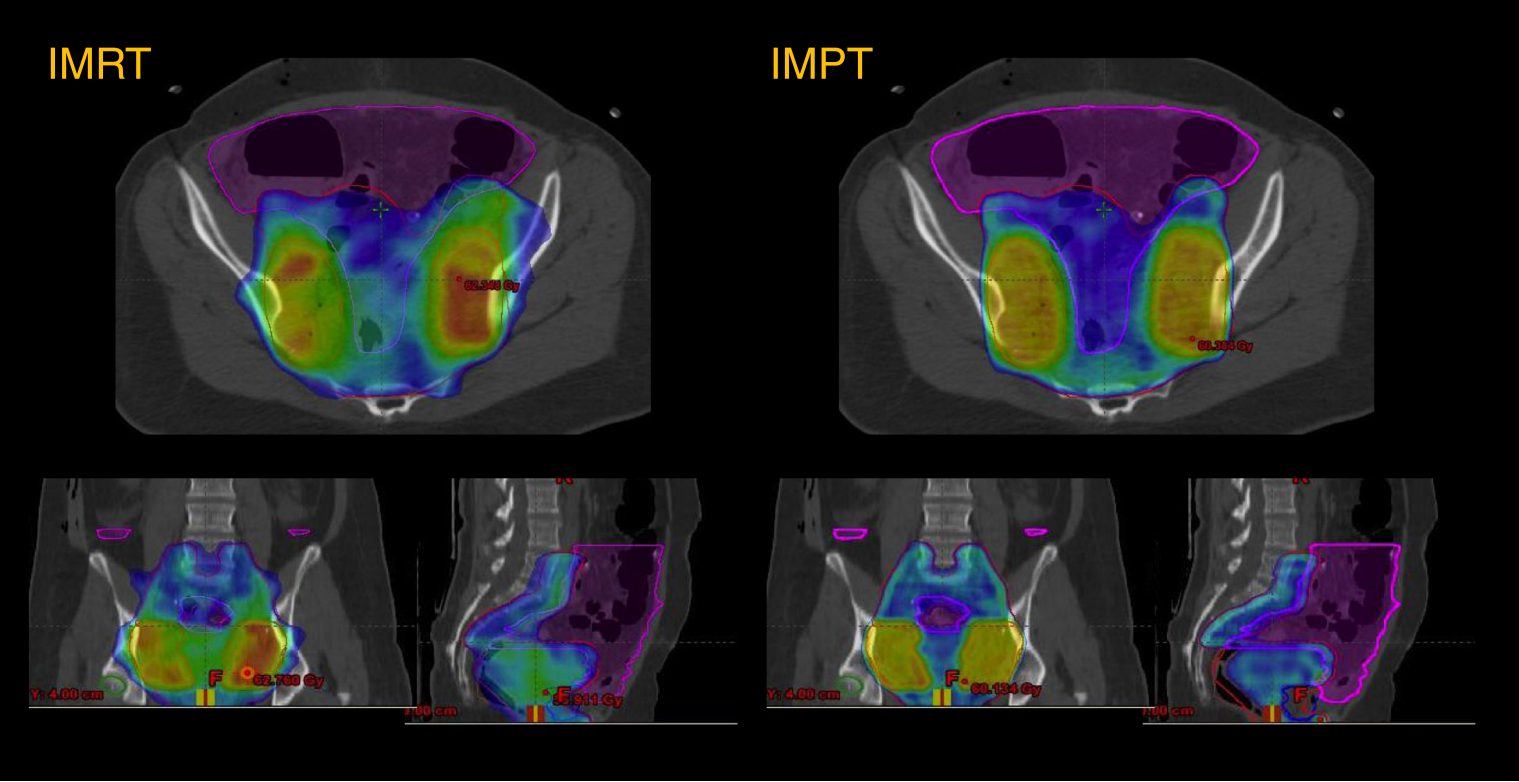
Example of Conformity and homogeneity of IMRT versus IMPT. Blue isodose = 95% isodose of PTV_A with 50.4 Gy prescribed dose; Yellow = 95% isodose of PTV_B with 59.36 Gy prescribed dose, Margenta: bowel contour.

All techniques fulfilled the defined the constraint for the PTVs D_95%_ > 95%. Detailed results are shown in Table 2. Although, differences were small, the best homogeneity for the PTV-A could be achieved with HT and IMRT for PEL and IMRT and VMAT for EFRT. Concerning PTV-B, IMPT created most homogeneous plans, followed by HT for PEL and IMRT for EFRT (as and example see Figure 1 with a comparison between IMRT and IMPT, similar qualitative results were obtained for the other techniques).

**Table 2:** **Summary of DVH analysis**

**Volume**	**Pelvic RT**				**EFRT**			
	**HT**	**IMPT**	**IMRT**	**VMAT**	**HT**	**IMPT**	**IMRT**	**VMAT**
**OAR Parameters**								
**Bowel V**_**10Gy**_ **(%)**	99.1 ± 1.3^a,b,c^	52.4 ± 15.8^d,e^	93.7 ± 4.1	94.6 ± 2.8	96.5 ± 4.3^a^	40.3 ± 7.1^d,e^	90.9 ± 6.6	94.0 ± 5.8
**Bowel V**_**20Gy**_ **(%)**	74.8 ± 12.6^a,c^	37.7 ± 13.3^d,e^	73.7 ± 12.0	74.2 ± 3.1	61.9 ± 1.4^a,c^	28.2 ± 7.0^d,e^	60.6 ± 9.3	59.8 ± 8.5
**Bowel V**_**30Gy**_ **(%)**	47.1 ± 18.8^a,c^	28.4 ± 10.6^d,e^	45.6 ± 12.9	44.8 ± 10.8	36.0 ± 10.6^a,c^	18.2 ± 5.5^d,e^	34.2 ± 8.5	32.7 ± 9.3
**Bowel V**_**50Gy**_ **(%)**	6.7 ± 3.6	5.6 ± 2.1^d,e^	11.8 ± 4.5	8.9 ± 2.5	6.5 ± 3.3^a^	2.9 ± 0.9^d,e^	8.8 ± 2.5	8.0 ± 2.8
**Bowel D**_**mean**_ **(Gy)**	27.6 ± 5.6^a^	18.6 ± 5.9^d,e^	30.2 ± 4.0	34.1 ± 7.0	24.0 ± 4.1^a^	13.8 ± 2.8^d,e^	26.3 ± 3.2	25.3 ± 3.7
**Rectum D**_**mean**_ **(Gy)**	47.8 ± 3.6^a^	41.4 ± 5.3^e^	47.2 ± 4.8	48.3 ± 1.6	48.2 ± 2.4^a^	41.3 ± 3.0^d,e^	48.5 ± 3.2	48.3 ± 1.5
**Bladder D**_**mean**_ **(Gy)**	49.2 ± 1.4^a^	41.6 ± 5.8^d,e^	49.1 ± 3.4	50.1 ± 1.6	48.4 ± 2.8^a^	40.8 ± 6.4^d,e^	49.5 ± 3.8	48.9 ± 2.3
**PTV Parameters**								
**HI PTV_A**	0.88 ± 0.02	0.87 ± 0.01	0.88 ± 0.01	0.87 ± 0.01	0.87 ± 0.01	0.87 ± 0.01	0.88 ± 0.01	0.88 ± 0.01
**HI PTV_B**	0.94 ± 0.01^a,b^	0.96 ± 0.01^d,e^	0.92 ± 0.01^f^	0.94 ± 0.01	0.94 ± 0.02^a^	0.96 ± 0.01^d,e^	0.93 ± 0.01	0.94 ± 0.01
**CN PTV_A**	0.88 ± 0.03^a,b^	0.92 ± 0.01^d,e^	0.82 ± 0.02^f^	0.88 ± 0.03	0.85 ± 0.13	0.88 ± 0.01^d^	0.82 ± 0.06	0.88 ± 0.03
**CN PTV_B**	0.87 ± 0.02	0.88 ± 0.05	0.84 ± 0.06	0.88 ± 0.03	0.86 ± 0.06^a,c^	0.89 ± 0.03^d^	0.78 ± 0.08	0.87 ± 0.08
**CI PTV_A**	1.09 ± 0.05^a,b,c^	1.03 ± 0.02^d,e^	1.20 ± 0.03^f^	0.98 ± 0.01	1.16 ± 0.07	1.05 ± 0.02^d,e^	1.22 ± 0.11^f^	0.97 ± 0.01
**CI PTV_B**	1.07 ± 0.06	1.08 ± 0.07	1.16 ± 0.09^f^	1.09 ± 0.05	1.15 ± 0.11	1.07 ± 0.06^d^	1.24 ± 0.13^f^	1.08 ± 0.10

HI: homogeneity index; CN: conformality number; CI: conformality index. V_xGy_: volume receiving at least x Gy. HT: helical tomotherapy; IMPT: intensity modulated proton therapy; IMRT: intensity modulated radiation therapy; VMAT: volumetric modulated arc therapy. EFRT: extended field radiation therapy. Statistical significance when p < 0.05. ^a^HT vs IMPT; ^b^HT vs IMRT; ^c^HT vs VMAT; ^d^IMPT vs IMRT; ^e^IMPT vs VMAT; ^f^IMRT vs VMAT.

**Figure 2:**
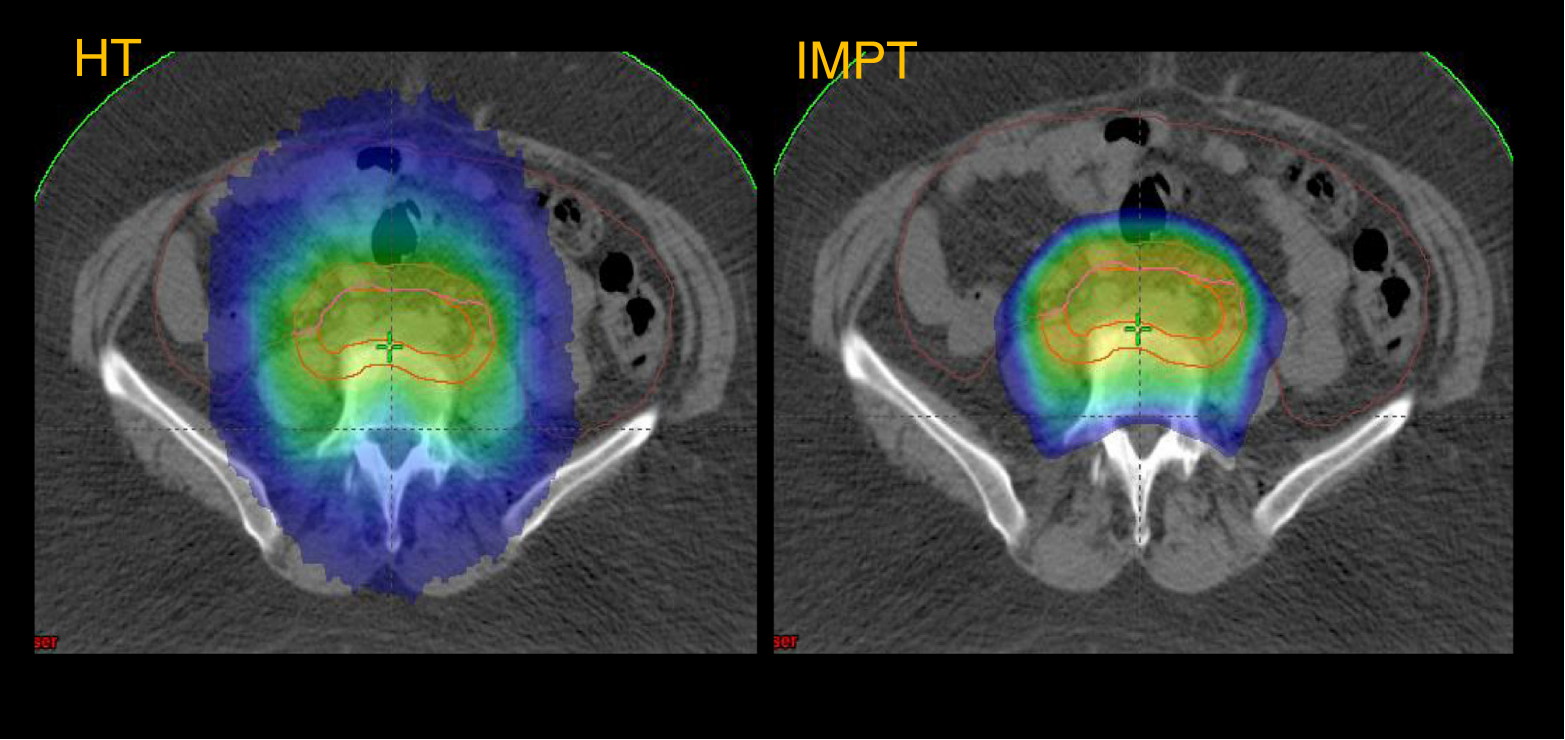
Example of Volume covered by 20 Gy (V_20Gy_, blue) for a patient with EFRT, HT vs IMPT.

**Figure 3:**
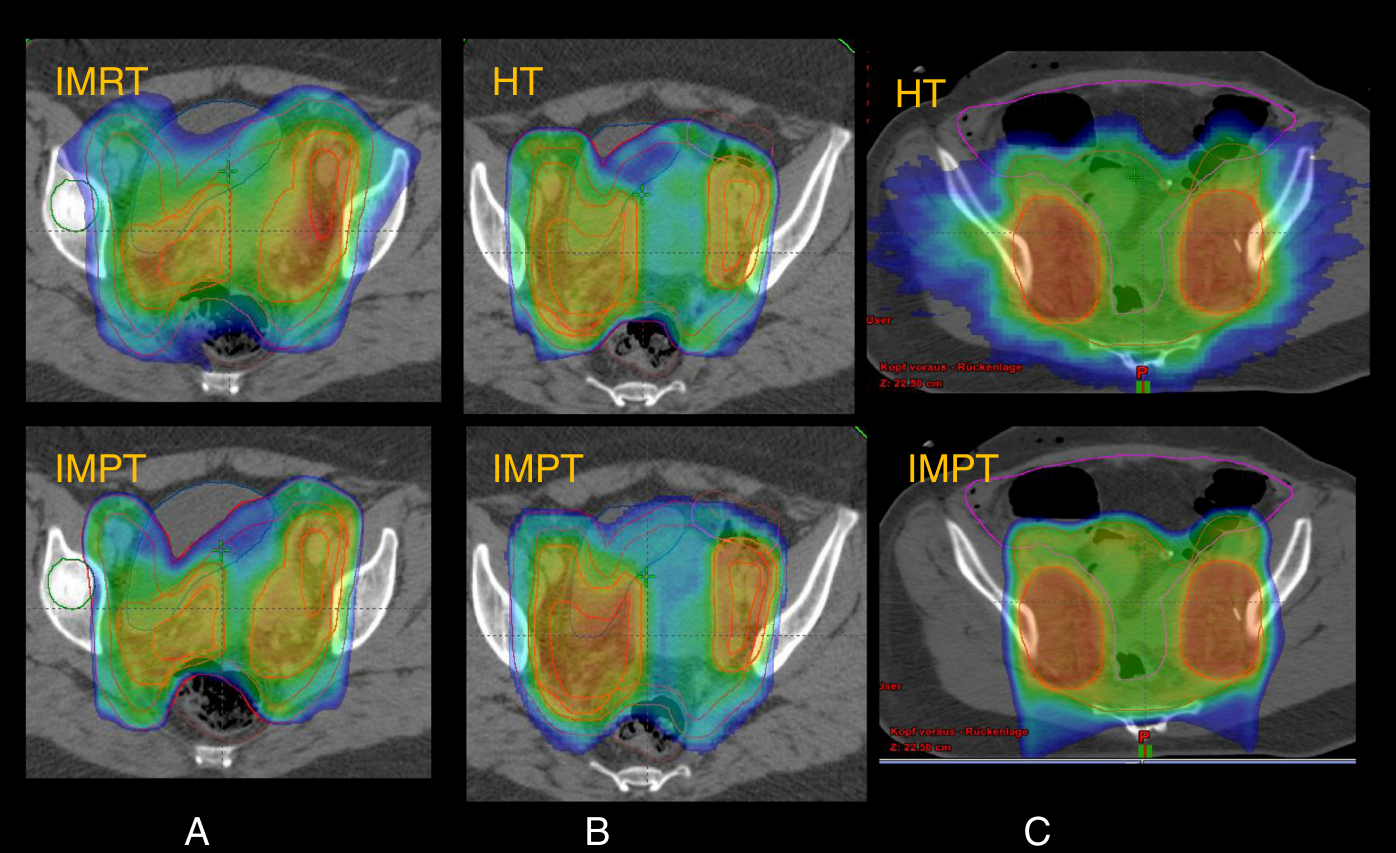
Examples of rectum and bowel sparing potential between techniques. **A** and **B**: rectum, colorwash is at 45 Gy; **B**: SB, colowash is at 30 Gy.

With IMPT, there was a significant reduction of the mean dose (D_mean_) of the bowels from 30.2 ± 4.0 Gy (IMRT); 27.6 ± 5.6 Gy (HT); 34.1 ± 7.0 (VMAT) to 18.6 ± 5.9 Gy (IMPT) for pelvic radiation and 26.3 ± 3.2 Gy (IMRT); 24.0 ± 4.1 (HT); 25.3 ± 3.7 (VMAT) to 13.8 ± 2.8 Gy (IMPT) for patients with EFRT, which corresponds to a reduction of 38-52% for the D_mean_ (bowels), (examples are shown in Figures 2 and 3). Futhermore, the low dose bath (V_10Gy_) to the bowels was reduced by 50% with IMPT in comparison to all photon techniques. Looking at the findings in terms of absolute volumes, all techniques allowed to maintain the high dose irradiation of the bowels to acceptable levels. The maximum bowel involvement at 50 Gy was less than 190 cc (96% confidence level) for IMRT and much less for all other technques in the PEL group. In the EFRT group, this ranged from about 400 cc for IMRT and VMAT to 150 cc for IMPT with averages ranging from 280 cc to 90 cc.

Furthermore, D_mean_ to the bladder and rectum was decresed by 7-9 Gy with IMPT in patents with pelvic radiation and EFRT. The dose to the high dose volume of the bowels could be reduced by IMPT, more pronounced for the EFRT than for PEL (Table 2, Figure 2).

The dose to the kidneys is not reported in detail since, for all optimisation engines, it was possible to achieve for all patients, organ sparing well below the assumed tolerance level of 30% for V_15Gy_ also in the more challanging case of the patients with EFRT.

## Discussion

Proton beam therapy has been used for various indications. Its physical characteristic Bragg peak allows for reducing the integral radiation dose to the normal tissue and selective sparing of certain organs of risk while providing an equal or superior target coverage. Furthermore, proton radiotherapy has the potential to permit dose escalation [17-20,30]. Few data is available with regard to cervical cancer treatment with different indications, concepts and proton techniques [14,21-23].

In contrast, our aim was to keep the small bowel dose as low as possible and we did not optimize plans in order to decrease the bone marrow dose. Since intracavitary brachytherapy delivered in conjunction with external beam radiotherapy has been an important component of the treatment of gynecologic cancers, GI toxicity is a major concern. The significant reduction of the normal tissue dose might result into a reduced risk of secondary malignancies in these younger patients [22,31].

Our aim was to compare all available highly sophisticated photon techniques like IMRT, helical tomotherapy or volumetric modulated arc therapy with intensity modulated protons in patients with pelvic and/or extended field treatments for cervical cancer.

Milby [22] published a dosimetric comparison of intensity-modulated radiotherapy with photons, only to the pelvic lymph nodes in combination with either passive scattering proton therapy IMPT or modulated photons to the para-aortic nodal region in women with locally advanced gynecologic malignancies. Georg [21] evaluated in a group of five patients with EFRT all these techniques. According to our results, proton and photon techniques were comparable in terms of homogeneity, conformity and coverage. Small, non-significant differences were seen with regard to homogeneity, conformation number and conformity index in favor for intensity modulated protons in our patients for pelvic radiation and extended field radiation.

The most striking difference between proton and photon techniques was related to the organs at risk. Since we have focused on further reduction of the considerable gastro intestinal toxicity our goal was to decrease the mean dose and the high dose volume as much as possible. Proton therapy resulted into a significant reduction of either mean dose by 33%, 38% and 45% compared with tomotherapy, modulated photons and volumetric modulated arc therapy respectively. One can assume that such a reduction of D_mean_ to the small bowel leads to a decrease in therapy related toxicity. In accordance to our data, Milby [22] found that all plans maintained excellent coverage of the planning target volume while decreasing the V_20Gy_, V_30Gy_, V_35Gy_, and V_40Gy_ of the small bowel. The volume covered by 50 Gy was reduced with protons in our patients and the low dose bath to the bowels (V_10%_) could be nearly halved with protons, both more pronounced for EFRT than for pelvic radiation. The rectal D_mean_ could be reduced by 7-9 Gy. Georg [21] reported on comparable rectal (wall) doses, while Milby [22] did not emphasize this. Relating to the therapeutic concept of external beam radiotherapy and brachytherapy in the treatment of cervical cancer the further dose reduction to the rectum is desirable and contributes to less rectal toxicity [32]. Although bladder dose was not a focus of this study, intensity modulated protons reduced the D_mean_ to the bladder by 7-9 Gy. The impact on toxicity is not clear, because no clear dose response has been defined until now. Renal toxicity, per se a factor of potential concern, was not identified as a relevant issue in this study since, also for the patients where the extension of the fields might involve these organs, sufficient protection was obtained by all the optimization engines. The quantitative tolerance considered for the study was V_15Gy_ < 30% which is more restrictive than what derived from the QUANTEC data [33] where this could be interpolated as V_15Gy_ < 40%.

One limitation of the present study is linked to the use of IMPT in the pelvis. Proton based intensity modulation with scanned beam technology can be affected by relevant range uncertainties (the uncertainty in the dose deposition with depth) because of the presence of bowel gas and/or bowel peristalsis. For these reasons, the clinical use of IMPT in the pelvis cannot be considered fully consolidated and detailed analysis of the impact of these dose uncertainties should be further explored. More in general, the use of IMPT with the inherent sharp dose fall-off, dosimetrically advantageous, shall be carefully considered and weighted against some obvious trade-offs (motion management, range uncertainty, delivery time, imaging and others). The IMPT are not necessarily to be considered the ultimate perfect technique to use but could be beneficial in some well selected challenging conditions. The present study constitute a theoretical investigation at planning level that might lead to deeper pre-clinical studies.

## Conclusion

Intensity modulated radiotherapy with photons, volumetric modulated arc therapy, helical tomotherapy and intensity modulated proton techniques were compared in cervical cancer patients. All techniques were proved to be dosimetrically adequate with regard to coverage, conformity and homogeneity. Intensity modulated protons offered the best sparing of the bowels and rectum and there for could contribute to a significant reduction of acute and late toxicity which should be proven in clinical trials.

## Abbreviations

CI: Conformity index
CN: Conformity number
CT: Computed tomography
CTV: Clinical target volume
DVH: Dose volume histogram
EFRT: Extended field radiotherapy
GI: Gastro-intestinal
GU: Genito-urinary
HDR: High dose rate
HI: Homogeneity index
HT: Helical tomotherapy
IMRT: Intensity modulated radiotherapy
IMPT: Intensity modulated proton therapy
MLC: Multileaf collimator
LN: Lymph node
MRI: Magnetic resonance imaging
OA: Oblique anterior
OAR: Organ at risk
PEL: Pelvic
PTV: Planning target volume
RA: RapidArc
RBE: Relative biological effectiveness
SIB: Simultaneous integrated boost
SB: Small bowel
TPS: Treatment planning system
VMAT: Volumetric modulated arc therapy
3D: Three dimensional

## References

[1] Morris M, Eifel PJ, Lu J, Grgsby P, Levenback C, Stevens R (1999). Pelvic radiation with concurrent chemotherapy compared with pelvic and para-aortic radiation for high-risk cervical cancer. N Engl J Med.

[2] Vale C, Tierney JF, Stewart LA, Brady M, Dinshaw K, Jakobsen A (2008). Reducing uncertainties about the effects of chemoradiotherapy for cervical cancer: a systematic review and meta-analysis of individual patient data from 18 randomized trials. J Clin Oncol.

[3] Green JA, Kirwan JM, Tierney JF, Symonds L, Collingwood M, Williams C (2001). Survival and recurrence after concomitant chemotherapy and radiotherapy for cancer of the uterine cervix: a systematic review and meta-analysis. Lancet.

[4] Kirwan JM, Symonds P, Green JA, Tierney J, Collingwood M, Williams C (2003). A systematic review of acute and late toxicity of concomitant chemoradiation for cervical cancer. Radiother Oncol.

[5] Varia MA, Bundy BN, Deppe G, Mannel R, Averette H, Rose P (1998). Cervical carcinoma metastatic to para-aortic nodes: extended field radiation therapy with concomitant 5-fluorouracil and cisplatin chemotherapy: a Gynecologic Oncology Group study. Int J Radiat Oncol Biol Phys.

[6] Grigsby PW, Lu JD, Mutch DG, Kim R, Eifel P (1998). Twice-daily fractionation of external irradiation with brachytherapy and chemotherapy in carcinoma of the cervix with positive para-aortic lymph nodes: Phase II study of the Radiation Therapy Oncology Group 92–10. Int J Radiat Oncol Biol Phys.

[7] Small W, Winter K, Levenback C, Iyer R, Gaffney D, Asbell S (2007). Extended-field irradiation and intracavitary brachytherapy combined with cisplatin chemotherapy for cervical cancer with positive para-aortic or high common iliac lymph nodes: results of ARM 1 of RTOG 0116. Int J Radiat Oncol Biol Phys.

[8] Rotman M, Pajak TF, Choi K, Clery M, Marcial V, Grigsby P (1995). Prophylactic extended-field irradiation of para-aortic lymph nodes in stages IIB and bulky IB and IIA cervical carcinomas. Ten-year treatment results of RTOG 79–20. JAMA.

[9] Portelance L, Chao KS, Grigsby PW, Bennet H, Low D (2001). Intensity-modulated radiation therapy (IMRT) reduces small bowel, rectum, and bladder doses in patients with cervical cancer receiving pelvic and para-aortic irradiation. Int J Radiat Oncol Biol Phys.

[10] Mundt AJ, Roeske JC, Lujan AE, Yamada S, Waggoner S, Fleming G (2001). Initial clinical experience with intensity-modulated whole-pelvis radiation therapy in women with gynecologic malignancies. Gynecol Oncol.

[11] Beriwal S, Gan GN, Heron DE, Selvaraj R, Kim H, Lalonde R (2007). Early clinical outcome with concurrent chemotherapy and extended-field, intensity-modulated radiotherapy for cervical cancer. Int J Radiat Oncol Biol Phys.

[12] Kavanagh BD, Pan CC, Dawson LA, Das S, Li X, Ten Haken R (2010). Radiation dose-volume effects in the stomach and small bowel. Int J Radiat Oncol Biol Phys.

[13] Chen MF, Tseng CJ, Tseng CC, Yu C, Wu C, Chen W (2008). Adjuvant concurrent chemoradiotherapy with intensity-modulated pelvic radiotherapy after surgery for high-risk, early stage cervical cancer patients. Cancer J.

[14] Isohashi F, Yoshioka Y, Mabuchi S, Konishi K, Koizumi M, Takahashi Y (2013). Dose volume histogram predictors of chronic gastrointestinal complications after radical hysterectomy and postoperative concurrent nedaplatin based chemoradiation therapy for early stage cervical cancer. Int J Radiat Oncol Biol Phys.

[15] Chopra S, Dora T, Chinnachamy A, Thomas B, Kannan S, Engineer R (2014). Predictors of grade 3 or higher late bowel toxicity in patients undergoing pelvic radiation for cervical cancer: results from a prospective study. Int J Radiat Oncol Biol Phys.

[16] Song WY, Huh SN, Liang Y, White G, Nichols R, Watkins W (2010). Dosimetric comparison study between intensity modulated radiation therapy and three-dimensional conformal proton therapy for pelvic bone marrow sparing in the treatment of cervical cancer. J Appl Clin Med Phys.

[17] van de Water TA, Lomax AJ, Bijl HP, de Jong M, Schilstra C, Hug E (2011). Potential benefits of scanned intensity-modulated proton therapy versus advanced photon therapy with regard to sparing of the salivary glands in oropharyngeal cancer. Int J Radiat Oncol Biol Phys.

[18] Weber DC, Wang H, Cozzi L, Dipasquale G, Khan H, Ratib O (2009). RapidArc, intensity modulated photon and proton techniques for recurrent prostate cancer in previously irradiated patients: a treatment planning comparison study. Radiat Oncol.

[19] Zhang X, Li Y, Pan X, Xiaogiang L, Mohan R, Komaki R (2010). Intensity-modulated proton therapy reduces the dose to normal tissue compared with intensity-modulated radiation therapy or passive scattering proton therapy and enables individualized radical radiotherapy for extensive stage IIIB non-small-cell lung cancer: a virtual clinical study. Int J Radiat Oncol Biol Phys.

[20] Boehling NS, Grosshans DR, Bluett JB, Palmer M, Song X, Amos R (2010). Dosimetric comparison of three-dimensional conformal proton radiotherapy, intensity-modulated proton therapy, and intensity-modulated radiotherapy for treatment of pediatric craniopharyngiomas. Int J Radiat Oncol Biol Phys.

[21] Georg D, Georg P, Hillbrand M, Poetter R, Mock U (2008). Assessment of improved organ at risk sparing for advanced cervix carcinoma utilizing precision radiotherapy techniques. Strahlenther Onkol.

[22] Milby AB, Both S, Ingram M, Lin L (2010). Dosimetric comparison of combined intensity-modulated radiotherapy (IMRT) and proton therapy versus IMRT alone for pelvic and para-aortic radiotherapy in gynecologic malignancies. Int J Radiat Oncol Biol Phys.

[23] Kagei K, Tokuuye K, Okumura T, Ohara K, Shioyama Y, Sugahara S (2003). Long-term results of proton beam therapy for carcinoma of the uterine cervix. Int J Radiat Oncol Biol Phys.

[24] Small W, Mell L, Anderson P, Creutzberg C, De Los Santos J, Gaffney D (2008). Consensus guidelines for delineation of clinical target volume for intensity-modulated pelvic radiotherapy in postoperative treatment of endometrial and cervical cancer. Int J Radiat Oncol Biol Phys.

[25] Toita T, Ohno T, Kaneyasu Y, Uno T, Yoshimura R, Japan Clinical Oncology Group (2010). A consensus-based guideline defining the clinical target volume for pelvic lymph nodes in external beam radiotherapy for uterine cervical cancer. Jpn J Clin Oncol.

[26] Gillin MT, Sahoo N, Bues M, Ciangaru G, Poenisch F, Arjomandy B (2010). Commissioning of the discrete spot scanning proton beam delivery system at the University of Texas M.D. Anderson Cancer Center, Proton Therapy Center, Houston. Med Phys.

[27] Arjomandy B, Sahoo N, Ciangaru G, Zhu R, Song X, Gillin M (2010). Verification of patient-specific dose distributions in proton therapy using a commercial two-dimensional ion chamber array. Med Phys.

[28] Feuvret L, Noel G, Mazeron JJ, Bey P (2006). Conformity index: a review. Int J Radiat Oncol Biol Phys.

[29] Mayo CS, Urie MM (2003). A systematic benchmark method for analysis and comparison of IMRT treatment planning algorithms. Med Dosim.

[30] Mock U, Bogner J, Georg D, Auberger T, Potter R (2005). Comparative treatment planning on localized prostate carcinoma conformal photon- versus proton-based radiotherapy. Strahlenther Onkol.

[31] Clivio A, Kluge A, Cozzi L, Koehler C, Neumann O, Vanetti E (2013). Intensity modulated proton beam radiation for brachytherapy in patients with cervical carcinoma. Int J Radiat Oncol Biol Phys.

[32] Georg P, Poetter R, Georg D, Lang S, Dimopoulos J, Sturdza A (2010). Dose effect relationship for late side effects of the rectum and urinary bladder in magnetic resonance image-guided adaptive cervix cancer brachytherapy. Int J Radiat Oncol Biol Phys.

[33] Dawson L, Kavanagh B, Paulino A, Shiva D, Miften M, Li A (2010). Radiation associated kidney injury. Int J Radiat Oncol Biol Phys.

